# Geospatial characteristics of medical workforce and infrastructure to combat COVID-19 in Kazakhstan: Natalya Glushkova

**DOI:** 10.1093/eurpub/ckac130.023

**Published:** 2022-10-25

**Authors:** N Glushkova, Y Semenova, B Jobalayeva, K Davletov, A Sarría-Santamera

**Affiliations:** 1 Department of Epidemiology and Biostatistics, Al-Farabi Kazakh National University, Almaty, Kazakhstan; 2 School of Medicine, Nazarbayev University, Nur-Sultan, Kazakhstan; 3 Department of Public Health, Semey Medical University, Semey, Kazakhstan; 4 Health Research Institute, Al-Farabi Kazakh National University, Almaty, Kazakhstan

## Abstract

**Background:**

After cessation of initial quarantine in Kazakhstan, the COVID-19 outbreak peaked in July 2020, imposing dramatic stress on the country's healthcare system. This study was focused on calculation of updated epidemiological characteristics, on evaluation of available medical workforce and infrastructure and the impact of workforce density on infected and dead individuals via ArcGIS platform.

**Methods:**

The national and local incidence rate (IR), mortality (M) and case-fatality rates (CFR) were calculated along with the population-weighted densities of beds, physicians, general practitioners, resuscitators, nurses and healthcare budget. Associations between the density of different health workers, infected and dead individuals were investigated using Poisson regression. Finally, we constructed vector maps of country regions clustered by IR and CFR to depict the density of beds and those health workers that were significantly associated with infection and death rates.

**Results:**

There is much heterogeneity between the country regions in terms of CFR (range from 0.28 to 2.57) and IR (range from 1.62 to 12.04), while density of beds was characterized by a relatively greater stability (range from 3.47 to 6.66) and so did density of physicians (range from 0.79 to 2.76) and density of nurses (range from 5.73 to 8.26). Densities of beds, physicians, general practitioners, resuscitators, and nurses have been linked significantly with infection and death rates.

**Conclusions:**

As COVID-19 epidemic is still far from ending, findings of this study could be of interest for policy makers to formulate an appropriate action plan in the view of possible repeated outbreaks.

**Key messages:**

- Available medical workforce and infrastructure were insufficient during the pandemic time in Kazakhstan.
- Densities of beds, physicians, general practitioners, resuscitators, and nurses are significantly associated with infection and death rates.

